# School performance and long-term outcomes of very preterm children conceived via *in vitro* fertilization

**DOI:** 10.5935/1518-0557.20190063

**Published:** 2020

**Authors:** Khalid Al-Hathlol, Omar Majed Al-Obaid, Thekra Solaiman Al-Gholaiqa, Bishayer Al-Hathlol, Abdullah Eid Abdulaal, Rafeef Ibrahim Al-Hajress, Futun Abdulrahman Al-Joufi, Nada Faris Al-Hassan, Abdulaziz Gassam Al-Otaibi

**Affiliations:** 1 Department of Pediatrics. King Abdullah International Medical Research Center / King Saud bin Abdulaziz University for Health Sciences - King Abdulaziz Medical City. Riyadh -Kingdom of Saudi Arabia

**Keywords:** children, IVF, outcome, preterm, school

## Abstract

**Objective:**

To assess the impact of *in vitro* fertilization (IVF) on school performance and long-term outcomes in very preterm children aged 8-16 years.

**Methods:**

Seventy-nine children born after IVF were compared with 79 randomly selected matched controls born after spontaneous conception (SC). Information was obtained from parents via a questionnaire administered through telephone interviews looking into school performance, including preschool education, repeated grades, extra lessons, special education needs, and learning difficulties; long-term status, including incidence of attention deficit hyperactivity disorder and autism; and family profile. Gross motor function was assessed against the gross motor function classification system based on information given by the subjects' families.

**Results:**

Mothers of IVF children were more likely to have a high educational level than mothers of SC children. Moreover, a greater proportion of IVF children had received preschool education than SC children. After adjusting for potential confounders, there was no difference in the school performance or long-term outcomes between IVF and SC children.

**Conclusion:**

In our study, the school performance and long-term outcomes of very preterm children born after IVF and of their spontaneously conceived peers were comparable. This information can help provide guidance to families and educators.

## INTRODUCTION

Assisted reproductive technology (ART) in the form of *in vitro* fertilization (IVF) has become widely applied in the treatment of couples with infertility. In general, IVF is a highly sophisticated procedure. It involves stimulating the ovaries with fertility hormones, retrieving eggs surgically from the ovaries, and fertilizing them with sperm in a laboratory dish. Then one or more embryos are transferred to the uterine cavity (ACOG, 2005; Harrison & Taylor, 2006). IVF technique has evolved significantly after the first successful IVF birth in 1978. The number of children born as a result of the procedure has been steadily increasing worldwide over the last 30 years (Andersen *et al*., 2008).

The developments in ART have significantly increased the rates of preterm birth, while advances in neonatal care have greatly improved the survival rates of very preterm infants over the last three decades (Jarjour, 2015). The World Health Organization (WHO) reported that across 184 countries, the rate of preterm birth ranges from 5% to 18% of babies born (World Health Organization, 2018).

It is well known that children born preterm have an increased risk of poor neurodevelopmental function compared with their peers born at term. These outcomes were related, in part, to the neonatal morbidities and, more importantly, to the developing brain being more susceptible to stress imposed by prematurity (Jarjour, 2015; Ahlsson *et al*., 2015). Likewise, in recent years several studies have raised concerns about the health outcomes of children born via ART. Several factors have been postulated to explain potentially adverse outcomes, including underlying parental infertility, procedural factors, and genetic disorders (Kallen, 2014).

Thus, preterm children of infertile couples are vulnerable to additional risk factors for adverse outcome, which may have a range of mental and motor consequences (Fortunato & Tosti, 2011; Connolly *et al*., 2010). Long-term functional problems are often reported for full-term children conceived via IVF in comparison with their naturally conceived peers (Bay *et al*., 2013; Bay, 2014). However, the published data concerning developmental outcomes in preterm children conceived via IVF are sparse (Ito *et al*., 2006; Xing *et al*., 2014; Ramoğlu *et al*., 2016); to date, there has been no study evaluating the educational and other long-term outcomes at school age, a highly challenging developmental stage, in very preterm children born after assisted reproduction, including IVF. The aim of this study was to compare the school performance and long-term outcomes of very preterm children conceived via IVF *versus* their naturally conceived peers.

## MATERIAL AND METHODS

### Participants

The study population comprised school-age children, born after IVF and spontaneous conception (SC) between January 2001 and December 2009, who were managed in the neonatal intensive care unit (NICU) at King Abdulaziz Medical City (KAMC), Riyadh, Kingdom of Saudi Arabia. The research ethics committee in the institution approved this study. The neonatal department consists of a level-III NICU with 40 beds that provides full neonatal intensive care and a level-II intermediate care nursery with 35 beds, both providing neonatal medical services to approximately 9,000 live-born infants every year. The units are run by seven full-time, board-certified neonatologists. Almost all pediatric subspecialties, including pediatric surgery, neurology, clinical genetics, and radiology, are readily available in the hospital. This academic center also includes an active IVF department that provides comprehensive reproductive management for many infertile couples. IVF is defined as a fertility treatment that involves removing eggs from the ovary, fertilizing them outside the body, and then implanting the fertilized eggs into the uterus. Intracytoplasmic sperm injection (lCSI) procedures were not included in this study.

Our study included very preterm birth (gestational age ≤32 weeks) and very low birth weight (VLBW; birth weight ≤1500 g) children admitted to the NICU. Children with major birth defects were excluded.

### Procedure and Measures

After obtaining parental consent for participation, SC school children were randomly selected and matched one to one with their IVF counterparts by age (8-16 years) and sex. Neonatal data, including birth weight, gestational age, and multiple births were obtained from the NICU database. Information on school performance, long-term health status, and family profile were obtained using a well-structured questionnaire administered during the telephone interviews conducted by trained staff, as well as from the available children's medical records.

The recorded study outcomes included the following: school performance, including preschool education, repeated grades, extra lessons, special education needs, and learning difficulties in reading, writing or math; long-term conditions such as attention deficit hyperactivity disorder (ADHD), autism, and visual or hearing impairment. The gross motor function of the children was assessed from the families' reports using the gross motor function classification system (GMFCS). The GMFCS has five functional levels ranging from 1 to 5. Children scoring 1 are typically normal and can walk without restrictions, while children scoring 2-5 usually have impaired motor function and individuals scoring 5 present significant impairment and limited self-mobility (Palisano *et al.,* 1997). The reliability of family report using this tool has been established for children aged between 6-12 and 12-18 years (Morris *et al.,* 2004). Family profile was also recorded; this included parental age and educational level, which was scored as low (elementary school), middle (secondary school), or high (university or higher level of education).

### Statistical analysis

The data were analyzed on software package SPSS (version 21.0; SPSS, Chicago, IL, USA). The IVF and SC groups were compared for individual characteristics and outcomes. Continuous variables were described as mean ± SD; categorical data were presented as numbers and proportions. The Chi-square test was used to compare categorical data, and Student's t-test was applied to compare continuous variables. Analyses yielding a value of *p* less than 0.05 were considered statistically significant. Multivariable logistic regression analysis was performed to assess the independent effect of IVF on school performance and long-term outcome among very preterm children. Adjustments were made for potentially confounding variables, including gestational age, multiple births, maternal education level, and preschool education.

## RESULTS

A total of 107 IVF-conceived children aged 8-16 years were identified from the NICU database. Of these, 28 children were excluded from analysis for the following reasons: no response to several phone calls (10 children); parents declined to join the study (9 children); and presence of major birth defects (9 children). The remaining 79 children were compared with 79 randomly selected matched controls born after spontaneous conception.

The baseline characteristics of children and parents in the IVF and SC groups are presented in Table 1. Compared with SC children, the IVF children were born at a higher gestational age (*p*=0.04) and were significantly more involved in multiple births (*p*=0.01). Other variables including birth weight, sex distribution, and current age were comparable between the matched groups.

Analysis of parental data showed that mothers of IVF children had higher levels of education than mothers of SC children (*p*=0.03), although other variables, including mother age at birth and parental age at the time of the study did not differ significantly between the groups (Table 1).

**Table 1:** Child and parental baseline characteristics of the IVF and SC study groups

Variable	IVF (*n*=79)	SC (*n*=79)	*p* value
Children current age (years)	12.3±2.4	12.3±2.4	1.00
Children age range (years)	8-16	8-16	1.00
Children sex distribution			1.00
Boys	40 (51)	40 (51)	
Girls	39 (49)	39 (49)	
Gestational age (weeks)	29.1±2.3	28.3±2.2	0.04
Birth weight (grams)	1128±260	1073±230	0.17
Multiple birth	70 (89)	10 (13)	0.01
Maternal age at birth (years)	30±4.2	30±4.9	0.76
Current maternal age (years)	42.±4.1	42±5.3	0.78
Current paternal age (years)	46±3.8	46±4.7	0.75
Maternal education level			0.03
Low	2 (3)	9 (11)	
Middle	55 (70)	57 (72)	
High	22 (27)	13 (17)	
Paternal education level			0.34
Low	1 (1)	2 (3)	
Middle	55 (70)	57 (72)	
High	23 (29)	20 (25)	

IVF: *in vitro* fertilization,

SC: Spontaneous conception.

Data presented as *n* (%) or mean ± standard deviation.

Table 2 shows the school performance and long-term outcomes of the IVF and SC groups. A significantly greater proportion of IVF children received preschool education compared with SC children (*p*=0.01). There were no statistically significant differences in the numbers of children with repeated grades, learning difficulties, extra lessons or special education needs between the IVF and SC children. Likewise, the numbers of children with outcomes such as ADHD, autism, visual impairment, hearing impairment, or impaired gross motor function were similar in both groups.

**Table 2:** School performance and long-term outcomes in the IVF and SC study groups

Variable	IVF (*n*=79)	SC (*n*=79)	*p*value
Preschool education	47 (59)	25 (32)	0.01
Repeated grade	5 (6.3)	9 (11)	0.26
Learning difficulties	12 (15)	19 (24)	0.16
Extra lessons	20 (25)	16 (20)	0.45
Special education need	2 (2.5)	5 (6.3)	0.24
ADHD	9 (11)	5 6.3)	0.26
Autism	2 (2.5)	1 (1.3)	0.56
Visual impairment	10 (13)	14 (18)	0.37
Hearing impairment	2 (2.5)	4 (5.1)	0.41
Impaired gross motor function	3 (3.8)	2 (2.5)	0.65

IVF:* in vitro* fertilization,

SC: Spontaneous conception,

ADHD: attention deficit hyperactivity disorder.

Data presented as *n* (%).

After adjusting for confounding variables, multivariable analysis revealed no statistically significant difference in the school performance or long-term outcomes between the IVF and SC groups (Table 3).

**Table 3:** Association of IVF conception with school performance and long-term outcome in very preterm children

Variable	OR	95% CI	*p*value
Repeated grade	0.45	0.11 – 2.49	0.36
Learning difficulties	0.63	0.17 - 2.33	0.49
Extra lessons	0.74	0.19 - 2.76	0.65
Special education need	2.93	0.27 – 30.8	0.37
ADHD	2.56	0.48 - 13.4	0.26
Autism	2.14	0.11 - 57.4	0.65
Visual impairment	1.12	0.27 - 4.34	0.90
Hearing impairment	3.25	0.27 - 38.7	0.35
Impaired gross motor function	1.12	0.53 - 23.6	0.92

IVF: *in vitro* fertilization,

OR: odds ratio,

CI: confidence interval.

ADHD: attention deficit hyperactivity disorder.

## DISCUSSION

Research worldwide has frequently shown concern for school performance and the long-term outcomes of survivors of prematurity. Nonetheless, the combined long-term effects of both IVF and prematurity have not been assessed among school-age children (Moreira *et al*., 2014). Our study revealed that very preterm children born after IVF are not at higher risk of educational limitations or adverse long-term outcomes compared with children born after natural conception.

To the best of our knowledge, this study is the first to look at the combined effects of IVF and prematurity on educational and long-term outcomes for school-age children. A few studies have reported long-term outcomes during the preschool period for very preterm children born after ART. Ito *et al*. (2006) reported on the neurodevelopmental outcomes in very preterm children during the preschool period. However, the pattern of intellectual function may change as a child grows older, thus deficits in cognitive functions may only be detectable in later childhood (Basatemur & Sutcliffe, 2008). Furthermore, different selection criteria were applied, thus yielding variable results. For instance, children born after ovulation induction have been included in the analysis of cohorts of children conceived via ART; in addition, both preterm and full-term children were included in the same analyses (Ito *et al*., 2006; Xing *et al*., 2014; Ramoğlu *et al*., 2016).

As this is the first study describing the school performance and other long-term outcomes in preterm children born via IVF, a direct comparison with outcomes of similar studies of IVF children born at term is challenging. Mothers of IVF children tended to be more highly educated than SC mothers, which may have influenced the cognitive function of their children (Mahalingaiah *et al*., 2011; Agarwal *et al*., 2005; Leslie *et al*., 2003). The study findings revealed that school performance remained comparable between IVF and SC children after correction for differences in maternal education and other variables, such as gestational age and multiple births.

Many studies have investigated the long-term effects of IVF in children born at term*.* The majority of results have been reassuring, showing no increased risk of neurodevelopmental disorders such as autism and ADHD, or motor disabilities in IVF children compared with children conceived naturally; our results were consistent with these reports (Hart & Norman, 2013a; 2013b). Considering the double tension of prematurity and IVF therapy, we believe that our findings provide families and professionals in health and educational services with supportive information concerning the educational and long-term health status of very preterm children conceived via IVF. Our findings might also be used as baseline information to help design follow-up studies.

One limit to the generalizability of our results was the relatively small sample size, which in part reflects the refusal of some IVF parents to participate for social reasons. In addition, GMFCS was used as a standardized system to assess motor function, while other study outcomes were assessed by telephone interview only. Assessment of academic performance through objective indicators might enhance the reliability of measurement in further research. Another point to consider is that the study population was limited to IVF-conceived children.

The inclusion of children born following other forms of ART, including intracytoplasmic sperm injection (ICSI), should be considered in future research to obtain more consistent information.

In conclusion, we found that the school performance and long-term outcomes of very preterm children born via IVF treatment and of their naturally conceived peers were similar. Hence, our findings may help provide guidance for families and professionals in health and educational services. Our data may also be used as the basis for designing follow-up studies.
